# MiR-140 inhibits classical swine fever virus replication by targeting Rab25 in swine umbilical vein endothelial cells

**DOI:** 10.1080/21505594.2020.1735051

**Published:** 2020-02-29

**Authors:** Panpan Xu, Shuangkai Jia, Kai Wang, Zhixin Fan, Hongqing Zheng, Jiangman Lv, Yanfen Jiang, Yufeng Hou, Bihao Lou, Hongchao Zhou, Yanming Zhang, Kangkang Guo

**Affiliations:** a College of Veterinary Medicine, Northwest A&F University, Yangling, Shaanxi China; b Medical College of QingHai University, Xining, Qinghai, China

**Keywords:** Classical swine fever virus, MIR-140, Rab25, 3ʹ UTR, inhibition

## Abstract

Classical swine fever virus (CSFV) is one of the most important viral pathogens leading worldwide threats to pig industry. MicroRNAs (miRNAs) play important roles in regulating virus replication, but whether miRNAs affect CSFV infection is still poorly understood. In previous study, we identified four miRNAs that were down-regulated by CSFV in swine umbilical vein endothelial cells (SUVEC). In this study, miR-140, one of the most potently down-regulated genes was investigated. We found that the miRNA expression was significantly inhibited by CSFV infection. Subsequent studies revealed that miR-140 mimics signiﬁcantly inhibited CSFV replication, while the inhibition of endogenous miR-140 enhanced CSFV replication. By using bioinformatics prediction, luciferase reporter system, real-time fluorescence quantitative PCR (RT-qPCR) and Western blot assays, we further demonstrated that miR-140 bind to the 3ʹ UTR of Rab25 mRNA to regulate its expression. We also analyzed the expression pattern of Rab25 in SUVECs after CSFV infection. The results showed that CSFV infection induced Rab25 expression. Finally, Rab25 was found to promote CSFV replication. In conclusion, this study demonstrated that CSFV inhibits miR-140 expression and miR-140 inhibits replication by binding to host factor Rab25.

## Introduction

Classical swine fever virus (CSFV) is an important member of *pestivirus genus* within the *Flaviviridae family* [1]. Classical swine fever (CSF) is one of the diseases notifiable to the World Organization for Animal Health and represents one of the leading worldwide threats to pig industry. Persistent CSFV infection is the leading cause of chronic CSF. Thus, elucidate the pathogenic mechanism of CSFV may largely help to develop effective prevention and control strategies.

Rab GTPases comprise the largest subfamily of small GTPases and play a master role in regulating intercellular vesicle and protein transport [2]. The mammalian genome encodes three Rab11 proteins, Rab11a, Rab11b and Rab11 c (Rab25) and change alternately from GTP- to GDP- [2,3]. The Ras small G protein superfamily have been identified as a key regulator of the intracellular transport system [4]. Rab25 is involved in transcytosis, endocytic sorting, and transport [5]. Currently, it has been reported that the aberrant expression of Rab25 was linked to cancer development and Rab25 is recognized as an oncogene [6].

Small RNA (microRNA, miRNA), a regulatory molecule in cells [7,8] that participates in a variety of life activities, and are also involved in the interaction of host cells with virus [9]. miRNAs are abundant in many types of mammalian cells [10] and appear to target about 60% of human and mammals genes [11,12]. Many miRNAs are evolutionarily conserved, which implies that they have important biological functions [13,14]. Accumulating studies showed that miRNAs participated in both the virus infection process and the host immune response [9,15]. The host-source miRNAs can directly or indirectly regulate the gene expression of the virus, which play a regulatory role in the viral infection and replication. It has been proved that viral infection leads to significant changes in the expression pattern of miRNA in the host cell and the host-source miRNAs could further regulate host gene expression to combat virus infection. For instance, in human immunodeficiency virus (HIV) infection, the expression of miR-17-5p and miR-20a is down-regulated [16].

MiRNAs also can direct regulate virus replication. Previous study found that the expression of the host-source miR-32 could effectively inhibit the genome replication of human foamy virus (PFV). MiR-32 plays an antiviral effect by targeting the PFV genome open reading frame 2 (ORF2) [17]. Another way is that microRNAs affect the replication of virus by targeting the non-coding region of host protein gene [18–20]. In our previous study, we identified four miRNAs that were down-regulated by CSFV in swine umbilical vein endothelial cells (SUVEC) [21,22] and our unpublished data]. Herein, miR-140, one of the most potently down-regulated genes was investigated. We showed that miR-140 inhibited the replication of CSFV. We further demonstrated the interaction between miR-140 and Rab25. It provides not only new clues to understand the interactions between host and virus but also new potential antiviral strategies for developing anti-CSFV treatment in the future.

## Materials and methods

### Cells and viruses

Swine umbilical vein endothelial cells (SUVEC) were established and preserved by laboratory and were cultured as previously described [23]. Human embryonic kidney (HEK-293 T) cells were stored in our laboratory. SUVEC and HEK-293 T cells were cultured in high glucose Dulbecco’s-modified Eagle’s medium (DMEM, Gibco, UK) supplemented with 10% fetal bovine serum (FBS, Gibco, UK), 100 IU/ml penicillin, and 100 mg/ml streptomycin at 37°C, 5% carbon dioxide (CO_2_) incubator. CSFV Shimen strain (GenBank: AF092448) was obtained from the China Institute of Veterinary Drug Control (Beijing, China) and was propagated in PK15 cells as described [24,25].

### Plasmid construction

To construct the pmirGLO targets luciferase reporter plasmid (P0198; Promega, Madison, Wisconsin, USA), the 3ʹ UTR of Rab25 were cloned using PCR of cDNA of SUVEC, then cloned into the 3ʹ UTR of the *Renilla* luciferase gene using the *Sal*I and *Xho*I restriction sites of the pmirGLO vector. Rab25 3ʹ UTR (5ʹ-CTGTGGT-3ʹ and 5ʹ-CTTGGT-3ʹ) were mutated to be their complementary sequence before artificial synthesis. Then, the oligonucleotides were synthesized, annealed into double strands, and cloned into the psiCheck-2 vector. The constructed plasmids were named as pmirGLO-Rab25-WT and pmirGLO-Rab25-MUT. To construct the overexpression vectors, the CDS of Rab25 were amplified using PCR from the SUVEC cDNA, then cloned into pEGFP-C1 and pDSRed1-N1 vector. To construct the inference vectors, using online design software (http://rnaidesigner.thermofisher.com/rnaiexpress/) and selecting high score sequence; then, the oligonucleotides were synthesized, annealed into double strands, and cloned into the pCDH-CMV-513B-U6 vector. The primer sequences are shown in Table 1.

**Table 1.** Sequences of primers, miRNAs, and siRNAs used in this study.

Primer	Sequence (5ʹ-3ʹ)	Use
miR-140-F	CUCAACUGGUGUCGUGGAGUCGGCAAUUCAG	To generate miR-140,
miR-140-R	CCACAGGGUAGAACCACGGA	Overexpression of miR-140
miR-140-inhibitor	CUGAAUUGCCGACUCCACGACACCAGUUGAG	Silencing of miR-140
U6-F	CTCGCTTCGGCAGCACA	qPCR for detection of U6
U6-R	AACGCTTCACGAATTTGCGT	
Rab25-F	GTGTCCAAGCAGAGGCAGAA	qPCR for detection of Rab25
Rab25-R	ACAGGGTCGGTGCTAGATGA	
CSFV-F	GATCCTCATACTGCCCACTTAC	qPCR for detection of CSFV
CSFV -R	GTATACCCCTTCACCAGCTTG	
β-actin-F	CAAGGACCTCTACGCCAACAC	qPCR for detection of β-actin
β-actin-R	TGGAGGCGCGATGATCTT	
NS2-F	GGAAAGATAGATGGCGGTTG	Establishment of a standard curve for CSFV
NS2-R	TCTAAGCACCCAGCCAAGG	
Rab25-3ʹ UTR-F	CGAGCTCCCTCATCTAGCACCGACC	To generate pmirGLO-Rab25-3ʹ UTR
	sac1	
Rab25-3ʹ UTR-R	GCTCTAGACTCAGAAGGACTGTGAACAGG	
	xba1	
Rab25-sh1	GGGAAGACCAATCTGCTATCC	siRNA to knockdown Rab25
Rab25-sh2	GGTGTTTGACCTAACCAAGCA	

### Plasmid and miRNA transfection

miRNA mimics and inhibitors listed in Table 1 were synthesized by RiboBio (Guangzhou, China). For miR-140 mimics, inhibitors or control, 8 × 10^4^ cells were seeded in a 12-well plate and transfection with riboFECT™ CP Reagent following manufacturers’ instructions. The recombinant plasmid, pEGFP-Rab25, was transfected with Turbofect (Thermo, USA) according to the manufacturers’ instructions.

### Prediction of miRNA-mRNA target by online software

RNA22 V2 and RNAhybrid online software were used to predict the ability of miR-140 seed region targeting Rab25 3ʹ UTR. First, their sequences were edited into the FASTS format, and the sequence of them was introduced into the software, and then the corresponding conditions were set before the alignments. In the process of using software, the main consideration was a few aspects: (1) whether the free energy of binding between miRNA and target gene 3ʹ UTR was within −20 kcal/mol. (2) compare the conservation of miRNA seed sequence among different species. (3) whether the miRNA seed region had a strict base pair complement with the 3ʹ UTR of target gene.

### Luciferase reporter assay

For luciferase assays, 2 × 10^4^ cells/well were seeded in 48-well plate and transfected with the pmirGLO-Rab25-WT, pmirGLO-Rab25-MUT and a negative control using transfection reagent (R0531, Thermo, USA). At 48 h post-transfection, cells were lysed and luciferase activity was examined using the dual-luciferase reporter assay system (E1910, Promega, USA) according to the manufacturers’ instructions. All experiments were performed in triplicate and the results were expressed as *ﬁreﬂy* luciferase activity normalized to *Renilla* luciferase activity.

### RNA extraction and quantitative reverse transcription PCR (RT-qPCR)

Total RNA was extracted from cells or cell culture supernatant using RNAiso Plus (TaKaRa, Dalian, China), following manufacturer’s instructions. The RNA samples were reverse into cDNA using the MiX–x™ miRNA First strand synthesis kit and prime Script™RT reagent Kit using gDNA Eraser (TaKaRa, Dalian, China), respectively. The abundance of the miRNA and RNA were determined by Quantitative PCR (qPCR) using speciﬁc primers (Table 1) with SuperReal PreMix Color (SYBR Green) (TIAN GEN, Beijing, China) and U6 (Table 1) was used as control. Then, Rab25 and CSFV mRNA were determined by RT-qPCR using specific primers (Table 1) with SuperReal PreMix Color (SYBR Green) (TIAN GEN, Beijing, China), and β-actin was used as control. Relative expression was analyzed using the comparative threshold cycle (Ct) method.

To determine CSFV RNA copy numbers, the fragment of the CSFV NS2 was amplificated by using specific primers (Table 1). Standard curves were generated from serial ten-fold dilutions of the NS2 templates. CSFV genomic RNA copies in culture supernatant were calculated by normalization to the standard curve.

### Western blot analysis

SUVEC were seeded in a 6-well plate. At 48 h post-transfection or CSFV infection, the cells were collected. For Western blot, the lysed cells were prepared and subjected to 12% SDS-polyacrylamide gel electrophoresis and transferred to a polyvinylidene fluoride (PVDF) membrane (RoChe). The membrane was blocked with 5% milk-TBS-Tween 20 for 2 h at room temperature. The membrane was blocked with the polyclonal anti-Rab25 antibody (CSB-PA019175LA01HU, cusabio), and monoclonal anti-β-actin antibody (AB0035, Abways). Incubating the primary antibodies for 12 h at 4°C and secondary antibodies, horseradish peroxidase (HRP) goat anti-rabbit IgG (H + L) (A21020-1, abbkine), for 2 h at room temperature. The blots were developed using enhanced chemiluminescence reagents (Genshare, Xi’An, China).

### Statistical analysis

Statistical analysis was performed using GraphPad Prism version 5.0 (GraphPad Software, San Diego, CA, USA) and significant differences between the two groups were determined using two-tailed Student’s t-tests. P < 0.05 was considered statistically significant.

## Results

### MiR-140 is down-regulated by CSFV infection

**Figure 1.**
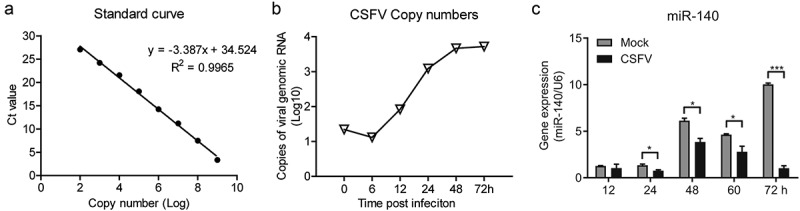
CSFV infection down-regulates miR-140 expression. (a) The standard curve of for detecting CSFV RNA genome. (b) Quantification of CSFV viral genomic RNA in supernatant. The copy number of CSFV viral genomic RNA is calculated by using the standard curve in (a). Cells were seed in a 48-well plate and infected with CSFV (MOI = 3). Then, the supernatant was collected at 0, 6, 12, 24, 48, and 72 h post-infection. (c) Real-time qRT-PCR analysis of miR-140 expression (normalized to U6 expression). The SUVECs were infected with CSFV (MOI = 0.1) and were collected at 12, 24, 48, and 60 h post-infection. Results are expressed as mean ± SD of three independent experiments. p values were calculated using Student’s t-test. *P < 0.05, ***P < 0.001.

The copy number of CSFV in cell supernatant was quantified and growth curve of CSFV was generated for the selection of optimal time-points. Thus, the absolute quantitative standard curve was established first (Figure 1(a)). Next, the cells were seeded in a 48-well plate and infected with CSFV at a multiplicity of infection (MOI) of 3. The CSFV genomic RNA in cell supernatant was quantified by RT-qPCR at different time points after infection. The results showed that CSFV replication reached the platform stage at 48 h post-infection (Figure 1(b)).

Previous data showed that miR-140 was down-regulated after CSFV infection in SUVECs. To verify this, SUVEC were infected with CSFV at a 0.1 MOI and the miR-140 expression level was measured at different time post-infection. The results showed that miR-140 expression was down-regulated in CSFV infected group compared to Mock (un-infected) group (Figure 1(c)). In particular, at 24, 48, 60, and 72 h post-infection, the miR-140 expression was significantly attenuated upon CSFV infection. Taken together, these results demonstrated that CSFV infection down-regulate the miR-140 expression in SUVECs.

### MiR-140 inhibits CSFV replication

**Figure 2.**
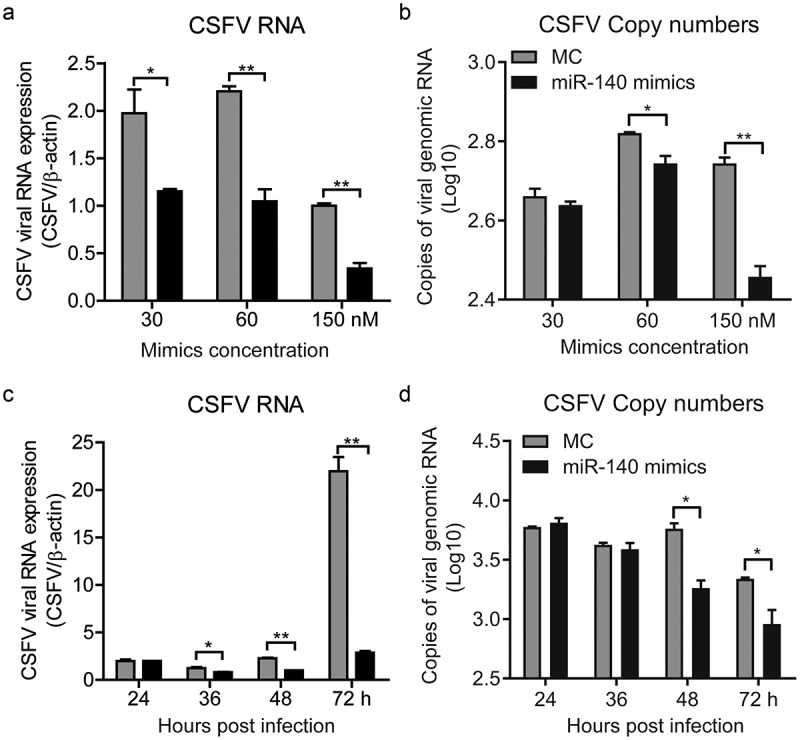
miR-140 mimics inhibits CSFV replication. (a and b) SUVEC were transfected with various concentrations of miR-140 mimics (30, 60, and 150 nM) or mimic control (MC) and infected with CSFV (MOI = 0.1). (a) Real-time qRT-PCR analysis of CSFV viral RNA (normalized to β-actin expression) at 48 h post-infection. (b) Quantification of CSFV viral genomic RNA in supernatant. The copy number of CSFV viral genomic RNA is calculated by using the standard curve in (Figure 1(a)). (c and d) SUVEC were transfected with of miR-140 mimics (60 nM) or mimic control (MC) and infected with CSFV (MOI = 0.1). The sample was collected at 24, 36, 48, and 72 h post-infection. (c) Real-time qRT-PCR analysis of CSFV viral RNA (normalized to β-actin expression). (d) Quantification of CSFV viral genomic RNA in supernatant. The copy number of CSFV viral genomic RNA is calculated by using the standard curve in (Figure 1(a)). Results are expressed as mean ± SD of three independent experiments. p values were calculated using Student’s t-test. *P < 0.05, **P < 0.01.

The miR-140 was down-regulated in SUVECs infected with CSFV, it is reasonable to speculate that miR-140 may have the ability to combat virus infection. To explore this, the miR-140 mimics was transfected into SUVECs and then the cells were infected with CSFV (MOI = 0.1). At 48 h post-infection, the CSFV genomic RNA of infected cells was quantified by RT-qPCR. As shown in Figure 2(a), the miR-140 mimics significantly down-regulated CSFV RNA amount with a dose-dependent manner, suggesting miR-140 could suppress CSFV replication. To further study the effect of miR-140 on CSFV, the viral genomic RNA in cell supernatants was also measured. The results showed miR-140 also potently reduced CSFV RNA amount in the cell supernatants (Figure 2(b)).

In order to further explore the effect of miR-140 on CSFV infection. SUVECs were transfected with miR-140 mimics (60 nM) and then infected with CSFV (MOI = 0.1). The CSFV RNAs were quantified at different time point post infection in cells and supernatants, respectively. The results showed that the CSFV was significantly down-regulated in both infected cells and supernatant from 48 h post-infection (Figure 2(c,d)). This study showed that miR-140 inhibits CSFV replication in a dose-dependent and time-dependent manner.

**Figure 3.**
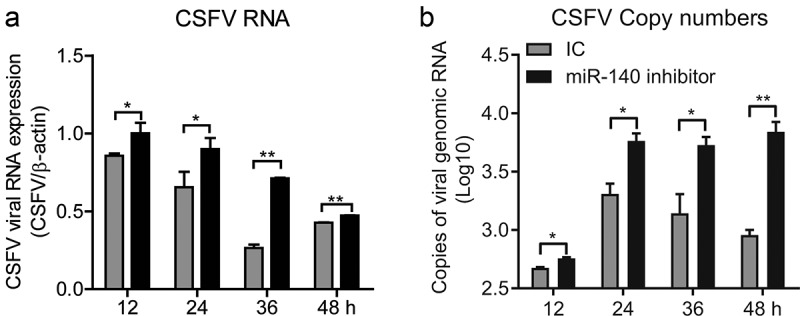
miR-140 inhibitor promotes CSFV replication. SUVECs were transfected with miR-140 inhibitors (200 nM) or inhibitor control (IC) and infected with CSFV (MOI = 0.1). (a) Real-time qRT-PCR analysis of CSFV viral RNA (normalized to β-actin expression) at 12, 24, 36, 48 h post-infection. (b) Quantification of CSFV viral genomic RNA in supernatant at 12, 24, 36, 48 h post-infection. The copy number of CSFV viral genomic RNA is calculated by using the standard curve in (Figure 1(a)). Results are expressed as mean ± SD of three independent experiments. p values were calculated using Student’s t test. *P < 0.05, **P < 0.01, ***P < 0.001.

The SUVECs were transfected with miR-140 inhibitor (200 nM) and then infected with CSFV (MOI = 0.1). The CSFV RNAs were quantified at 12 h, 24 h, 36 h, and 48 h post-infection in both infected cells and supernatant. The results showed that the CSFV RNA was significantly up-regulated in cells from 12 h after infection, but in cell supernatant was from 24 h after infection (Figure 3(a,b)).

### MiR-140 targets to the 3ʹ UTR of Rab25

**Figure 4.**
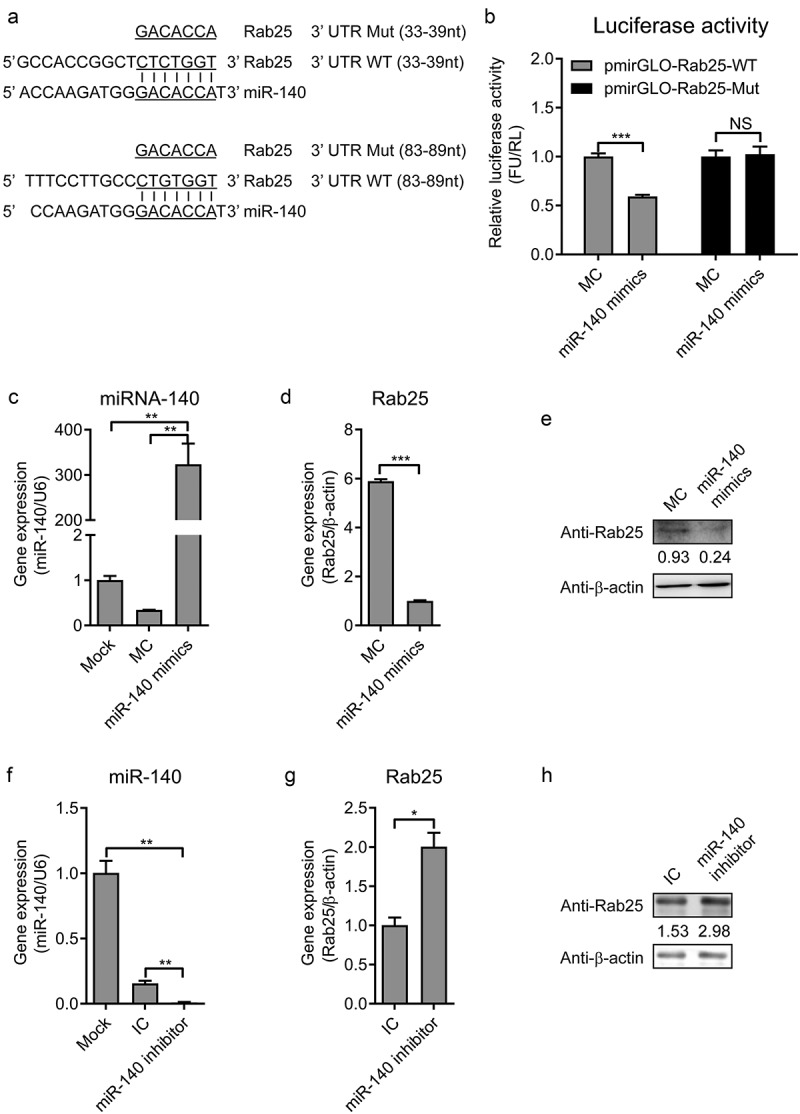
MiR-140 binds to the 3ʹ UTR of Rab25. (a) RNA22 V2 and RNAhybrid online software were used to verify the target gene of miR-140. Alignments showed that there were two complementary sequences between miR-140 sequences and Rab25-3ʹ UTR sequences. Underlined sites indicate miR-140 seed region and mutated sequences are shown. (b) Dual-luciferase assays. Wild-type (WT) (pmirGLO-Rab25-WT) or mutant (pmirGLO-Rab25-Mut) luciferase-reporter vector and miR-140 mimics were co-transfected into cells. Luciferase activity were determined at 48 h post-transfection by dual-luciferase assays. (c, d, and e) 60 nM miR-140 mimics or mimics control (MC) were co-transfected into cells. (c) Real-time qRT-PCR analysis of miR-140 expression (normalized to U6 expression) at 48 h post-infection. (d) Real-time qRT-PCR analysis of Rab25 expression (normalized to β-actin expression) at 48 h post-infection. (e) Immunoblot analysis of Rab25 protein levels at 48 h post-infection. The number means the intensity of Rab25 protein level (top) normalized to that of β-actin. (f, g, and h) 60 nM miR-140 inhibitor or inhibitor control (IC) were co-transfected into cells. (f) Real-time qRT-PCR analysis of miR-140 expression (normalized to U6 expression) at 48 h post-infection. (g) Real-time qRT-PCR analysis of Rab25 expression (normalized to β-actin expression) at 48 h post-infection. (h) Immunoblot analysis of Rab25 protein levels at 48 h post-infection. The number means the intensity of Rab25 protein level (top) normalized to that of β-actin. Results in (a, c, d, f, and g) are expressed as mean ± SD of three independent experiments. p values were calculated using Student’s t-test. Western blots were analyzed and quantified using the Image J software. *P < 0.05, **P < 0.01, ***P < 0.001.

The RNA22V2 and RNAhybrid online software were used to predict the target gene of miR-140 in cells. The Alignments results identified that there were two complementary sequences of miR-140 and Rab25 3ʹ UTR. The first one locates at 33 ~ 39 nt of Rab25 3ʹ UTR with one mispairing and the second locates at 83 ~ 89 nt of Rab25 3ʹ UTR with completely complementary sequences (Figure 4(a)). To confirm that the seed sequence of miR-140 binds to the Rab25 3ʹ UTR, we generated two *firefly* luciferase reporter gene constructs: a pmirGLO-Rab25-WT containing the predicted target sites in the 3ʹ UTR and a pmirGLO-Rab25-Mut, where the seed nucleotides of the target sites were mutated. When miR-140 binds to the pmirGLO-Rab25-WT construct, the luciferase activity will be down-regulated since the base pairing occurs between the seed region of miR-140 and the target site. The pmirGLO-Rab25-WT or pmirGLO-Rab25-Mut construct was co-transfected with miR-140. As shown in Figure 4(b), miR-140 significantly inhibited the *firefly* luciferase activity of the 3ʹ UTR-WT, while this was not observed in the mutated construct, revealing a specific binding between miR-140 and Rab25.

To further determine the capability of miR-140 binds to Rab25 3ʹ UTR, miR-140 mimics transfected into cells and the expression of Rab25 was measured by RT-qPCR and western blot. As shown in Figure 4(c), the up-regulation of miR-140 was confirmed. As expected, the gene expression and protein expression level of Rab25 was also significantly reduced in the miR-140 mimics transfected cells (Figure 4(d,e)). Conversely, the Rab25 expression was significantly activated by miR-140 inhibitor (Figure 4(f) to 4 H). Taken together, these results demonstrated the miR-140 binds to Rab25 to inhibit its expression in SUVECs.

### CSFV infection down-regulates miR-140 and up-regulates Rab25 expression

**Figure 5.**
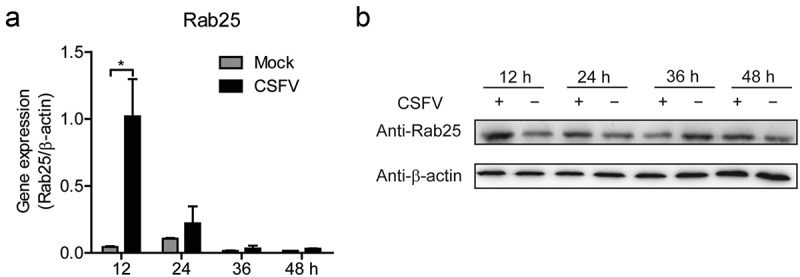
The expression of Rab25 is upregulated after CSFV infection in SUVECs. (a) Real-time qRT-PCR analysis of Rab25 expression (normalized to β-actin expression) at 12 h, 24 h, 36 h, and 48 h after CSFV infection in SUVEC. (b) Immunoblot analysis of Rab25 protein levels. The treatment is the same as (b). Results in (a) were expressed as mean ± SD of three independent experiments. p values were calculated using Student’s t-test. Western blots were analyzed and quantified using the Image J software. *P < 0.05.

In this study, we found that the miR-140 expression was inhibited by CSFV infection (Figure 1(d)). In addition, we also revealed that miR-140 inhibits CSFV replication and Rab25 expression in SUVECs. However, whether CSFV infection affects Rab25 expression was still unknown. To explore this, SUVEC was infected with CSFV at an MOI of 0.1. At certain times after infection, the expression level of Rab25 was studied at mRNA and protein level. The results showed that the Rab25 was up-regulated upon CSFV infection. In particular, the mRNA expression of Rab25 was significantly induced by CSFV infection at 12 h post-infection (Figure 5(a)). Consistently, a similar result was obtained by detecting Rab25 protein expression (Figure 5(b)). Together, we showed CSFV infection down-regulates miR-140, and up-regulates Rab25 expression at the early stage of viral infection.

## Rab25 promotes CSFV replication

**Figure 6.**
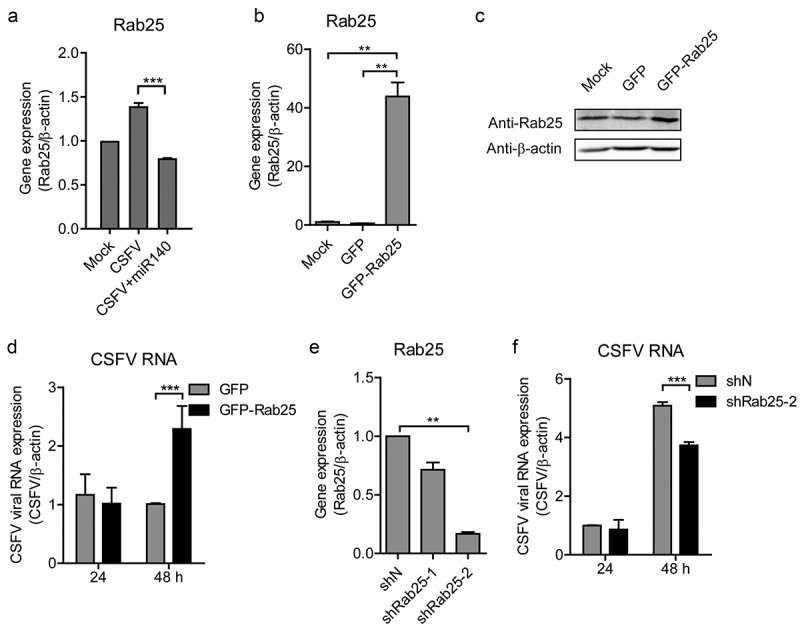
Rab25 promotes CSFV replication. (a) Real-time qRT-PCR analysis of Rab25 expression (normalized to β-actin expression) after transfection miR-140 mimics (150 nM) and infected with CSFV (MOI = 0.1). (b) Real-time qRT-PCR analysis of Rab25 expression (normalized to β-actin expression) after transfection of Rab25-GFP or control plasmid (GFP) for 24 h in SUVECs. (c) Immunoblot analysis of Rab25 protein levels. The number means the intensity of Rab25 protein level (top) normalized to that of β-actin. The treatment is the same as (a). (d) The SUVECs were transfected with Rab25-GFP or control plasmid and then infected with CSFV (MOI = 0.1). Real-time qRT-PCR analysis of CSFV viral RNA (normalized to β-actin expression) 24 h and 48 h post-infection. (e) The Rab25 gene was silenced by using shRNA approach. Real-time qRT-PCR analysis of Rab25 expression (normalized to β-actin expression) after transfection of shRab25 (shRab25-1, shRab25-2) or control (shN) plasmid. (f) The SUVECs were transfected with shRab25 (shRab25-1, shRab25-2) or control (shN) plasmid and then infected with CSFV (MOI = 0.1). Real-time qRT-PCR analysis of CSFV viral RNA (normalized to β-actin expression) 24 h and 48 h post-infection. Results in (A, C, D, and E) are expressed as mean ± SD of three independent experiments. p values were calculated using Student’s t-test. Western blots were analyzed and quantified using the Image J software. **P < 0.01, ***P < 0.001. NS, not significant.

To further assess the interaction of miR-140, Rab25, and CSFV infection, we measured the Rab25 expression in CSFV-infected miR-140-transfected cells. Although CSFV infection induced Rab25 expression, the Rab25 expression was decreased in CSFV-infected miR-140-transfected group (Figure 6(a)). To determine Rab25 regulate CSFV replication in cells, the recombinant plasmid pEGFP-Rab25 was transfected into SUVECs and then infected with CSFV at an MOI of 0.1. The overexpression of Rab25 was confirmed by RT-qPCR and Western blot (Figure 6(b,c)). Interestingly, the CSFV RNA level was significantly increased in the Rab25-overexpressed cells (Figure 6(d)). To further investigate the effect of Rab25 on CSFV replication, the Rab25 gene was silenced by using shRNA approach. The efficient knockdown was confirmed by RT-qPCR to measure the mRNA level of Rab25 (Figure 6(e)). Then, the control cells (shN) and Rab25 knockdown cells (shRab25-2) were both infected with CSFV at an MOI of 0.1. The result showed that the down-regulation of Rab25 expression significantly attenuated CSFV replication level at 48 h post-infection (figure 6(f)). Together, these results indicate that Rab25 supports the replication of CSFV and this might mediate the anti-CSFV potential of miR-140.

## Discussion

miRNAs are a kind of regulatory non-coding RNA that exist in eukaryotes and viruses. MiRNAs regulate virus by combining the 3ʹ UTR of the target genes to with degrade or inhibit their expression [8,26]. Cellular miRNAs play important roles in regulating virus replication. It has been reported that miR-135a regulates Hepatitis C virus (HCV) replication by targeting C-X-C motif chemokine ligand 12 (CXCL12) and receptor-interacting serine/threonine kinase 2 (RIPK2) [27]. MiR-30 c promotes porcine reproductive and respiratory syndrome (PRRSV) replication by targeting interferon-alpha/beta receptor beta chain (IFNAR2) [28]. In addition, miR-122 repress porcine circovirus type 2 (PCV2) by down-regulating the expression of nuclear factor of activated T-cells 5 (NFAT5) and aminopeptidase puromycin sensitive (NPEPPS) [29]. However, little is known about the relation between miRNAs in CSFV infection.

Classical swine fever (CSF) is a highly pathogenic infectious disease caused by CSFV. It has a wide range of epidemic and high incidence [30]. The classical symptom is extensive systemic bleeding [8,31,32]. In this study, we had showed that the expression of miR-140 was obviously down-regulated after CSFV infection SUVEC. miR-140 has been reported to play important role in many cellular responses. Mitofusin 1 (Mfn1) has an important role in cardiomyocyte apoptosis and miR-140 inhibits Mfn1 expression by targeting the 3ʹ UTR [33]. miR-140 targets the CXC group of chemokine ligand 12 (CXCl12) and drosophila mothers against decapentaplegic protein 3 (SMAD3) in chondrocyte differentiation [34,35]. In addition, MiR-140 also can suppress liver tumor by targeting the 3ʹ UTR of DNA methyltransferase 1 (Dnmt1) [36]. Importantly, miR-140 can suppress NF-κB activity by regulating the expression of nuclear receptor coactivator 1 (NCOA1) and nuclear receptor-interacting protein 1 (NRIP1) [37]. However, a few researches have been investigated its effect on virus replication. In this study, we demonstrated that CSFV infection down-regulated the expression of miR-140. The effect of miR-140 on CSFV replication was also explored by transfecting miR-140 mimics and inhibitors. The results showed that overexpression of miR-140 inhibits CSFV replication and the inhibition of miR-140 promotes CSFV replication.

Rab proteins are a large class of regulatory proteins and have been proved to be involved in the process of infiltration and replication of CSFV. Rab1A takes part in particle assembly of CSFV [38] and Rab2 can promote CSFV replication [39]. In addition, Rab5 promotes NS4B induced complex formation and thereby increases CSFV replication [40]. However, the effects of Rab25 on CSFV replication have received little attention. Coincidentally, Rab25 was characterized as the target genes of miR-140 by prediction. Firstly, the miR-140 targeting Rab25 was proved by using double luciferase reporting system, RT-qPCR and Western blot approaches. Then, the data also showed that Rab25 could promote the proliferation of CSFV. In addition, we found that the expression of Rab25 was up-regulated after CSFV infected cells. All these data showed that miR-140 inhibited CSFV replication by targeting Rab25 mRNA 3ʹ UTR. These data suggested that miR-140 inhibits the CSFV replication through the interaction with Rab25. At present, there is very little research regarding the regulation of CSFV in miRNAs. We speculate that Rab25 also has similar regulatory functions since Rab25 is found located in the secretory pathway of the opposite Golgi and Golgi vesicles, and could regulate the transportation through cyclic endosomes [41]. However, this hypothesis needs further investigation.

In conclusion, in this study, we investigated the interaction between miR-140, Rab25 proteins with CSFV replication. The miR-140 inhibits CSFV replication through targeting Rab25 and Rab25 support CSFV replication. This study provides mechanism insight into miRNA-viral mRNA interaction and new ideas for controlling CSFV infection.
